# Prostate stereotactic body radiotherapy with simultaneous integrated boost: which is the best planning method?

**DOI:** 10.1186/1748-717X-8-228

**Published:** 2013-10-02

**Authors:** Alison Tree, Caroline Jones, Aslam Sohaib, Vincent Khoo, Nicholas van As

**Affiliations:** 1 Royal Marsden NHS Foundation Trust, Fulham Road, London SW3 6JJ, UK; 2 Institute of Cancer Research, Surrey SM2 5PT, UK

**Keywords:** Stereotactic body radiotherapy, Prostate cancer, Focal boost

## Abstract

**Background:**

The delivery of a simultaneous integrated boost to the intra-prostatic tumour nodule may improve local control. The ability to deliver such treatments with hypofractionated SBRT was attempted using RapidArc (Varian Medical systems, Palo Alto, CA) and Multiplan (Accuray inc, Sunnyvale, CA).

**Materials and methods:**

15 patients with dominant prostate nodules had RapidArc and Multiplan plans created using a 5 mm isotropic margin, except 3 mm posteriorly, aiming to deliver 47.5 Gy in 5 fractions to the boost whilst treating the whole prostate to 36.25 Gy in 5 fractions. An additional RapidArc plan was created using an 8 mm isotropic margin, except 5 mm posteriorly, to account for lack of intrafraction tracking.

**Results:**

Both RapidArc and Multiplan can produce clinically acceptable boost plans to a dose of 47.5 Gy in 5 fractions. The mean rectal doses were lower for RapidArc plans (D50 13.2 Gy vs 15.5 Gy) but the number of missed constraints was the same for both planning methods (11/75). When the margin was increased to 8 mm/5 mm for the RapidArc plans to account for intrafraction motion, 37/75 constraints were missed.

**Conclusions:**

RapidArc and Multiplan can produce clinically acceptable simultaneous integrated boost plans, but the mean rectal D50 and D20 with RapidArc are lower. If the margins are increased to account for intrafraction motion, the RapidArc plans exceed at least one dose constraint in 13/15 cases. Delivering a simultaneous boost with hypofractionation appears feasible, but requires small margins needing intrafraction motion tracking.

## Introduction

Dose escalation is known to improve biochemical control in prostate cancer [1-4] however at conventional fractionations, this is associated with an increase in toxicity [1-3]. Studies deriving the alpha-beta ratio for prostate cancer from low-dose rate brachytherapy and external beam treatments have suggested the alpha-beta ratio is possibly as low as 1.5 Gy [5-7]. It is hypothesized that we can exploit this unusual tumour characteristic to permit radiobiological dose escalation without increasing toxicity using fewer, larger doses of radiation (hypofractionation). Studies of moderate hypofractionation (up to 3.5 Gy per fraction) have shown toxicity which is, at worst, equivalent to standard fractionation [8-10] and there are some early suggestions of improved biochemical control [9,10]. Further data is eagerly awaited. Stereotactic body radiotherapy (SBRT) is an emerging technique for treating localized prostate cancer using 4–5 doses of 7 Gy or higher (36.25 Gy in 5 fractions is a commonly used dose). Results from both retrospective and prospective series show good biochemical outcomes in those with low and intermediate risk disease [11-14], which serves to reaffirm the concept of a low alpha/beta ratio for prostate cancer. Almost all the literature on prostate SBRT delivers these treatments on Cyberknife but there is increasing interest in using conventional linacs to deliver SBRT.

Studies of patterns of failure following conventionally fractionated external beam radiotherapy show that the area responsible for local recurrence is the dominant intraprostatic nodule in 89% [15] – 100% [16] of cases. By dose escalating the dominant nodule we should increase biochemical control whilst avoiding the increase in side effects seen with whole gland dose escalation.

SBRT using Cyberknife is capable of producing dose distributions that are similar to those achieved with HDR, with a rapid dose fall off and the ability to create heterogeneous dose distributions within the prostate [17]. The objective of this study is to achieve a heterogeneous dose distribution, with the high dose region (47.5 Gy) targeted to the dominant intraprostatic nodule, whilst maintaining a dose of 36.25 Gy to the whole prostate. We compared the two methods of SBRT delivery available to us at our institution – Cyberknife (using Multiplan planning software) and RapidArc arc therapy using Varian linacs.

## Materials and methods

### Patient selection

This study was prospectively approved by the Royal Marsden service evaluation committee. Fifteen patients with intermediate or high risk prostate cancer, who had received standard IMRT at our institution, and who had a dominant prostate disease nodule (DPDN) on their diagnostic MRI, were selected. All patients had standard bladder filling protocol (300 mls at 45 minutes prior to planning CT scan) and no routine bowel preparation prior to the radiotherapy planning scan.

### Volume definition

The diagnostic MRI scan was fused with the planning CT scan. The DPDN was contoured jointly by the oncologist and the radiologist using MRI (T2 and diffusion-weighted sequences were used to delineate the boost volume in all cases) and with reference to the sites of highest Gleason score described on pathology. The volume outlined formed the boost target volume.

The prostate and proximal seminal vesicles were outlined to form the CTV. Normal structures were delineated as per institutional protocol including delineation of the rectum as a solid structure extending from the anus to the recto-sigmoid flexure, and delineation of the bladder as a solid structure from bladder base to dome.

### Margins

Our institutional margins for prostate SBRT using Cyberknife are 5 mm except 3 mm posteriorly. This is based on the literature on prostate SBRT [11,12] and is concordant with those used in the current randomized PACE trial comparing SBRT with IMRT [18]. This margin is likely to account for subclinical extra-prostatic extension [19,20] and treatment delivery uncertainties. The prostate and seminal vesicles CTV was therefore expanded by 5 mm isotropically except 3 mm posteriorly to form the PTV(prostate) for the Cyberknife plans.

For the RapidArc plans (which used RapidArc version 8.6), to provide pure dosimetric comparison, the first plan used the same margins (5 mm/3 mm posteriorly) as we would use with Cyberknife to form the PTV(prostate). However, it is established that intrafraction motion during conventional fractionation requires a margin of 2-3 mm [21-24]. Therefore a second plan was constructed with PTV (prostate) margins of 8 mm/5 mm posteriorly which is likely to be sufficient to account for intra-fraction motion after set-up to gold fiducial markers before treatment.

No PTV margin was put around the boost region which was always entirely contained within the prostate CTV. Whilst a 0 mm margin for treatment delivery accuracy is unlikely to be achievable, as the area surrounding the boost was within the CTV prostate, the dose fall off around the boost was relatively slow, and we noted that in most cases at 2 mm from the boost, the mean dose was maintained above 47 Gy. The boost volume (with no margin) is hereafter called PTV (boost).

### Dose

**Figure 1:**
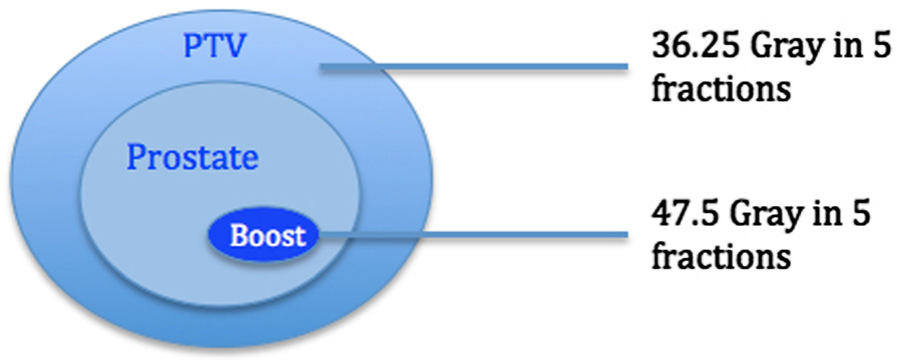
Planning schema.

Dose to the PTV(prostate) was 36.25 Gy in 5 fractions and dose to the PTV(boost) was 47.5 Gy. Dose schema is shown in Figure 1.

Plans for both RapidArc and Multiplan were normalized so that ≥95% of the target volume received 100% of the dose. The organ at risk (OAR) constraints were based on those currently used clinically at our institution (see Table 1) for prostate SBRT.

**Table 1:** Dose constraints for prostate SBRT planning

	**Rectum**					**Bladder**		
	**D50**	**D20**	**D10**	**D5**	**Hottest 1 cc**	**D40**	**D10**	**Hottest 10 cc**
Constraint (Gy)	18.1	29.0	32.6	36.25	37.3 (aim <36)	18.1	36.25	37.0

This planning study assumed that for Cyberknife plans treatment would be theoretically delivered with gold fiducial intra-fraction monitoring every 30–60 seconds. For RapidArc plans, it was assumed that patients would be set-up to gold fiducials prior to treatment.

Statistics were calculated using Prism version 6 (GraphPad software inc). Differences between planning methods were compared using a Wilcoxon matched-pairs signed rank test, as a Gaussian distribution could not be assumed and the correlation between 1 cc dose and various parameters (PTV overlap with rectum, distance from boost to rectum, PTV volume) was explored using linear regression.

## Results

### Plan demographics

Patients had a mean age of 72, with a mean PSA of 19.1 and a mean prostate volume of 57.4 cc. Seven patients had T3a disease and the remainder had T2a-T2c. Due to the time, and in 13/15 cases the use of hormonal therapy between the diagnostic MR and the planning CT scan, and the inherent differences in CT and MR prostate outlining there was often significant change between the two scans which made fusion sub-optimal. In these cases, the prostate contour on the side of the boost was prioritized so that the boost region remained in a representative position. The boost was in the left lateral position in 6 cases, the right lateral position in 5 cases, was centrally positioned in 2 cases and was centrally posterior in 1 case. The mean boost volume was 3.7 cc and the mean distance from the boost to the anterior rectal wall was 2.3 mm. The mean overlap between the PTV (5 mm/3 mm margin) and the rectum was 2 cc (range 0.02-4.26).

For Cyberknife plans the mean number of beams was 215 and the mean number of nodes was 59. The mean predicted treatment time was 46 minutes and the mean conformality index for the Cyberknife plans was 1.14. For RapidArc, the plans were constructed with a double arc, and took at mean of 5.9 minutes to deliver and had a mean conformality index of 1.04 (smaller margins) and 1.03 (larger margins). The median boost dose was 47.5 Gy for both planning methods but the mean D95 PTV(boost) was higher for Cyberknife (48.1 Gy) than RapidArc (47.4 Gy) and RapidArc with larger margins (46.9 Gy).

### Rectal doses

**Figure 2:**
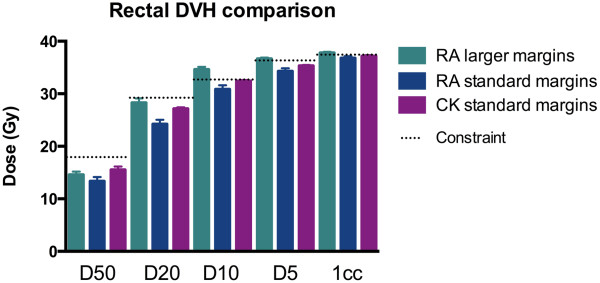
Mean rectal DVH data by planning method.

**Figure 3:**
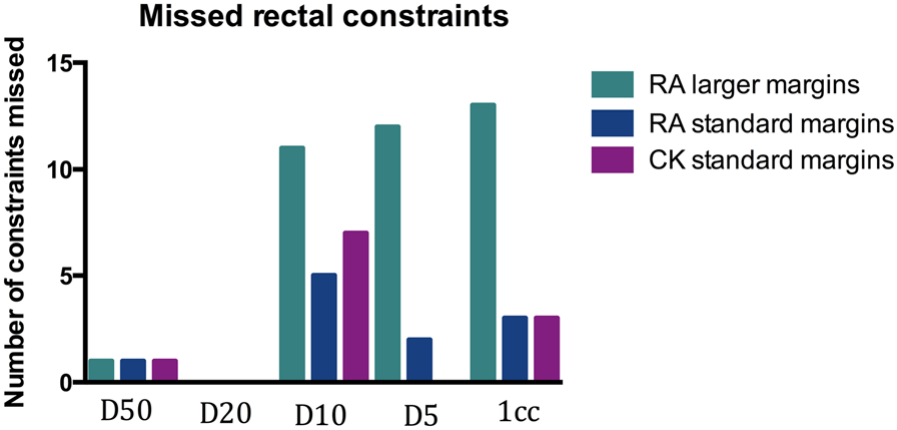
Number of missed rectal constraints by dose (43/75 for RapidArc larger margins, 11/75 for RapidArc standard margins, 11/75 for Cyberknife standard margins).

For the same margins, the mean doses to the rectum were higher on average for Multiplan (Figure 2 and Table 2) which may be partly a reflection of the way in which the optimization is driven in each system (e.g. we have found that with Version 8.6 of RapidArc the optimization is best if the OAR objectives are set much lower than the actual constraints, whereas with Multiplan over-reaching the possible OAR constraints can result in the degradation of the plan). In contrast, the number of constraints achieved vs failed was identical for Multiplan vs RapidArc with the same margins (see Figure 3). However for most cases where the constraint was failed, it was largely by less than 1 Gy. For example for the 12 plans which could not meet the D10 constraint of 32.6 Gy, 8 of achieved a D10 of 33.6 Gy i.e. only a 1 Gy relaxation of the constraint was needed for most plans to accomplish this. The constraint which was exceeded to the greatest extent was the rectal 1 cc dose (i.e. dose to the hottest 1 cc of rectum), which was in four cases > 2 Gy higher than the constraint (2 Rapid Arc plans, 2 Cyberknife plans).

**Table 2:** Rectal dose comparison (figures in bold have exceeded the constraint)

**Rectal dose**	**Margins**	**D50**	**D20**	**D10**	**D5**	**Hottest 1 cc**
**Multiplan**	5 mm/3 mm post	15.52	27.13	32.40	35.32	37.15
**RapidArc**	5 mm/3 mm post	13.17* (p = 0.01)	24.37* (p = 0.005)	30.86	34.29	36.78
**RapidArc larger margins**	8 mm/5 mm post	14.59	28.28	34.61* (p = 0.001)	36.63* (p = 0.0003)	**37.7**

*denotes those that are significantly different from Cyberknife plans (using a paired *t*-test, p values in brackets).

If the RapidArc margins were increased to 8 mm/5 mm posteriorly then 43/75 rectal constraints were missed. For example, the mean rectal D5 was 36.6 Gy for these plans (which is above the constraint of 36.2 Gy) and the mean rectal 1 cc was 37.7 Gy, (maximum constraint 37.3 Gy).

### Bladder doses

**Figure 4:**
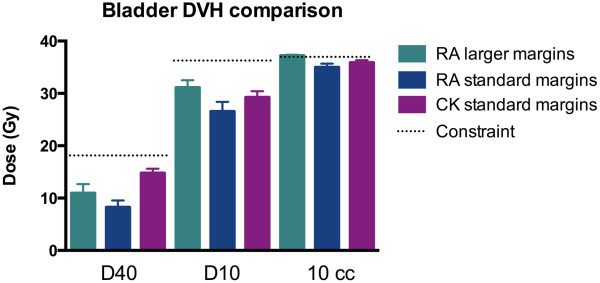
Mean bladder DVH data by planning method.

For the bladder constraints, Cyberknife plans had higher mean values for the D40, D10 and hottest 1 cc compared to the RapidArc plans with the same margins. For example the mean dose to the hottest 10 cc was 34.97 Gy for RapidArc and 35.88 Gy for Cyberknife. However, both of these are well within the constraint of 37 Gy (see Figure 4) and the number of missed constraints was similar.

The RapidArc plans with the larger margin gave more dose to all bladder constraints tested but they still met most constraints except the average dose to the hottest 10 cc of bladder which was 37.21 Gy, just outside the constraint. The association of bladder dose and genitourinary toxicity has not been well established with conventional fractionation and the implication of these dose differences in SBRT is not known.

### Are there factors which make focal dose escalation easier?

As the constraint to the hottest 1 cc of rectum was the hardest to meet, we examined whether certain factors are predictive of the value of this constraint. We investigated whether the extent of overlap between the PTV and the rectum, or the distance from the boost to the anterior rectal wall affected the ability to meet the most difficult constraint, the 1 cc rectum dose.

Surprisingly, neither of these factors are clearly correlated with the dose to the 1 cc rectum, except for the relationship between the Rectum-PTV overlap and the results of the RapidArc plans (p = 0.025). However this relationship loses statistical significance if the outlier (at 16 mm) is removed.

We also examined for any correlation between dose to the hottest 1 cc of the rectum and anterior-posterior, left-right patient separations at the level of the prostate and with prostate volume. No significant relationships were found.

## Discussion

Many planning studies have compared the ability of different planning systems to deliver SBRT. This study compares the ability of Cyberknife and RapidArc to achieve an intentionally heterogeneous dose distribution and thereby achieve a differential dose to two PTVs depending on risk of disease recurrence.

There are many logical reasons why dose-escalating the dominant tumour nodule may improve the therapeutic ratio. However this strategy relies on being able to delineate the dominant prostate disease nodule accurately. Newer MRI techniques have improved tumour delineation in prostate cancer [25-27].

In the detection of patients with prostate cancer, combined dynamic contrast-enhanced (DCE) or diffusion weighted (DWI) MRI detects a cancer-containing region of the prostate with a high sensitivity and specificity. Studies have reported sensitivities of 38-82% and specificities of 37–96% for DCE or DWI alone [25,26,28-30].

The combination of both DCE-MRI and DWI further improves delineation of tumour within the prostate [31-33]. The combination of DCE-MRI and MR-spectroscopy (MRS) may also improve tumour delineation [34]. It appears however that maximal tumour definition requires only 2 of the 3 modalities (DW-MRI, DCE-MRI and MRS), and that using all 3 does not improve prostate cancer detection [30].

Models which use MRI parameters (including DWI and DCE) on a voxel-by-voxel basis have been shown to be highly reliable for predicting tumour presence on pathology and suggested to be suitable for focal boost therapy [35]. The conspicuity of tumour nodules has been found to decrease in patients who have completed more than 3 months of androgen deprivation therapy (ADT) [36] and so it may be best to use a pre-ADT MRI to plan a focal boost, although the change in prostate size and shape over time may limit the accuracy of subsequent fusion to a radiotherapy planning CT. 18-F Choline-PET and Carbon-11 acetate PET have also been used to define a radiotherapy boost volume in planning studies [37,38].

Studies have attempted to model the extent of focal dose escalation feasible with conventional fractionation. Housri et al. [39] identified DPDNs in 24/42 men studied. In these men dose escalation to 151 Gy was possible in half the cases, and was more likely to be possible in those with a larger distance between the dominant nodule and the rectum, and in those with a smaller hip-to-hip distance on planning CT. This echoes our previously presented work, showing that DPDN within 1.5 mm of the rectum are more difficult to dose escalate [40]. However, in this series, distance to rectum did not correlate with rectal 1 cc dose, except for a finding of borderline significance for RapidArc plans. It may be that the ability to dose escalate to 47.5 Gy is correlated with distance to rectum, but that the dose to the 1 cc rectum has other more complicated determinants, including possibly a combined effect of the size of the PTV, bladder and rectum and size of the patient.

Both RapidArc and Multiplan are complex planning tools and the quality of a plan is likely to be dependent on the experience of the user. Whilst the plans in this paper represent the best plans our department could produce at the time, we have had longer experience with RapidArc and hence our Cyberknife plans may still reflect the steep gradient of our learning curve.

It may also be that with further experience we have learnt to improve the dose fall-off of our plans, facilitating high intraprostatic doses with less penalty in rectal dose constraints. In addition, this is a sample of 15 patients, and larger numbers may be needed to see significant correlations.

The two systems discussed here have markedly different treatment times. The rate at which dose is delivered is likely to have some implication however the size and direction of this influence is complex [41] and likely to have a differential effect depending on a/b ratio of the tissue in question. The oncological and side effect implications of a 40 minute vs a 6 minute treatment time, if any, are yet to be ascertained in vivo.

Both Multiplan and RapidArc planning systems can produce clinically acceptable plans which deliver a focal boost of 47.5 Gy in 5 fractions whilst treating the entire prostate to 36.25 Gy. The extent of focal dose escalation possible without unacceptable OAR dose penalty may seem surprising but the use of either many beams, or arcing beams facilitates a rapid dose fall-off. The Cyberknife system incorporates near real-time tracking of the prostate which allows smaller margins to be used, such as those used in this study, as it tracks and corrects for intra-fraction motion. This paper suggests that if intra-fraction motion could be tracked and corrected during arc-therapy on a conventional linac, then the dosimetry is likely to be at least as good as Cyberknife. However, without intra-fraction motion control, a margin of 2–3 mm is likely to be needed to account for intrafraction motion after initial set-up to gold markers [42] which in this study is associated with exceeding dose constraints around half of the time. Alternatively, if new flattening-free filter linacs can deliver these plans in much shorter times, then intra-fraction motion may be less important, although transient, significant excursions of prostate position are still possible [43].

The optimization algorithms for RapidArc and Multiplan are different and, at least in our hands, these differences result in a slightly lower mean dose for RapidArc compared to Multiplan. In further work, not shown here, we have found that Multiplan can produce similar DVH values to RapidArc on individual plans, once the lowest achievable levels have been established with a RapidArc plan. It may be that further improvement in the Multiplan plans is possible as we gain further experience with this technique or as the optimization algorithm evolves.

We have established that it is possible to focally dose escalate prostate SBRT to target the dominant lesion. The next step is to establish if this can be achieved in patients without significant increase in toxicity, and whether focal dose escalation translates into improved biochemical control. This is being tested with conventional fractionation in the FLAME trial [44] which is currently randomizing patients (single-blind) to standard radiotherapy (77 Gy in 35 fractions) or the same dose plus a simultaneous integrated boost to the dominant nodule to 95 Gy. This trial is defining the boost volume using MRI, including DCE- and DWI-MRI. The primary end-point is biochemical relapse-free survival at 5-years. Previous non-randomised studies have shown that moderate boosting to the dominant nodule (80–82 Gy) with conventional fractionation can be achieved without increase in toxicity [45-48].

The margins needed for an intra-prostatic boost are not well defined. Some studies have used 2-4 mm margins [45,47,49] whereas others have not used an intra-prostatic PTV margin [46]. As the boost is within the PTV (prostate) the dose fall-off is likely to be shallow enough to significantly dose-escalate 2-3 mm away from the delineated DPDN.

We have designed a pilot study of focal boosting using 5 fractions of SBRT, based on the planning technique used for this study. The SPARC trial (Stereotactic prostate augmented radiotherapy with Cyberknife) aims to establish if focal doses up to 47.5 Gy in 5 fractions (and 36.25 Gy in 5 fractions to the whole prostate) can be delivered to patients with intermediate and high risk prostate cancer without significant increase in acute toxicity.

## Conclusion

The ability to utilize non-coplanar, non-isocentric beams did not significantly improve focal boost plans compared to arc therapy. Rapid Arc plans delivered lower mean rectal doses, but both systems achieved the dose constraints in the same number of cases. However, without the ability to track intrafraction motion the PTV would have to be increased by a further 2-3 mm, particularly as the plans take an average of 5.9 minutes to deliver. If the PTV margin is increased to this extent, plans no longer meet the constraints in many cases. Intra-fraction motion monitoring facilitates the smaller margins needed to deliver a simultaneous integrated boost to the dominant prostate cancer nodule. We will shortly trial this technique clinically in a pilot study to assess whether these SIB plans can be delivered clinically without additional toxicity.

## Competing interests

Dr. van As is the chief investigator of a randomised clinical trial which is financially supported by Accuray inc.

Dr. Tree, Dr. van As and Dr. Khoo have received educational support grants to facilitate attendance at international conferences.

The other authors declare no conflicts of interest.

## Authors’ contributions

NVA, VK and AT developed the concept and design of this study. AT, AS and CJ participated in the contouring and/or radiotherapy planning. All authors helped to draft and approved the final version of the manuscript.
